# Occlusion myocardial infarction in type I NSTEMI: prevalence, characteristics, management, and long-term outcome

**DOI:** 10.1007/s00392-026-02966-8

**Published:** 2026-07-22

**Authors:** Lea Kirsten, Pedro Lopez-Ayala, Emel Kaplan, Jasper Boeddinghaus, Luca Koechlin, Lourdes Herraiz-Recuenco, Gabrielle Huré, Karin Wildi, Paolo Bima, Jonas Glaeser, Carlos C. Spagnuolo, Luca Crisanti, Òscar Miró, Michael Christ, Dagmar I. Keller, Javier F. Martin-Sanchez, Roland Bingisser, Gregor Leibundgut, Felix Mahfoud, Ivo Strebel, Christian Mueller, the APACE investigators, Desiree Wussler, Thomas Nestelberger, Maria Rubini Gimenez, Juliane Gehrke, Jude Formambuh, Tobias Zimmermann, Christian Puelacher, Ksenija Slankamena, Danielle M. Gualandro, Andreas Buser, Eliska Potlukova, Damian Kawecki, Nicolas Geigy, Katharina Rentsch, Beata Morawiec, Piotr Munzk, Beatriz Lopez-Barbeito, Gemma Martinez-Nadal, Carolina Fuenzalida, Sofia Calderón, Esther Rodriguez Adrada, Eva Ganovská, Jiri Parenica, Arnold von Eckardstein, Isabel Campodarve, Joachim Gea, Christian Nickel

**Affiliations:** 1 Cardiovascular Research Institute Basel (CRIB) and Department of Cardiology, University Hospital Basel, University of Basel, Basel, Switzerland; 2 Department of Cardiac Surgery, University Hospital Zürich, University of Zürich, Zurich, Switzerland; 3 Department of Intensive Care, Cantonal Hospital Aarau, Aarau, Switzerland; 4 Department of Medical Sciences, University of Turin, Turin, Italy; 5 Emergency Department, Hospital Clinic, IDIBAPS, University of Barcelona, Barcelona, Catalonia Spain; 6 Emergency Department, Kantonsspital Luzern, Lucerne, Switzerland; 7 Emergency Department, University Hospital Zurich, Zurich, Switzerland; 8 Servicio de Urgencias, Hospital Clínico San Carlos, Madrid, Spain; 9 Emergency Department, University Hospital Basel, Basel, Switzerland

**Keywords:** Occlusion myocardial infarction, Type I NSTEMI, Time to angiography, NSTEMI classification

## Abstract

**Background:**

The prevalence, characteristics, management, and long-term outcome of patients with non-ST-segment elevation myocardial infarction (NSTEMI) due to a completely occluded culprit coronary artery (NSTEMI-OMI) remain insufficiently characterised.

**Methods:**

In this international multicentre study, consecutive patients with centrally adjudicated type I NSTEMI were classified as NSTEMI-OMI if they had a TIMI flow grade of 0 or 1 or grade 2 with severe coronary narrowing (> 70%) and a high-sensitivity cardiac troponin T (hs-cTnT) ≥ 500 ng/L. Prospectively recorded chest pain characteristics, 12-lead ECG, serial hs-cTnT concentrations, time to coronary angiography/revascularisation, and 5-year mortality were compared in patients with NSTEMI-OMI versus other type I NSTEMI (NSTEMI-NOMI).

**Results:**

Among 801 patients with type I NSTEMI (median age 68 years, 22.2% female), 251 patients (31.3%) had NSTEMI-OMI. Patients with NSTEMI-OMI presented more often with persistent chest pain (56.1% vs. 37.9%, *p* < 0.001), more often had ST-segment depression (34.3% vs. 23.0%, *p* < 0.001), and exhibited higher and faster rising hs-cTnT concentrations. Time from admission to coronary angiography (median 7.2 h [IQR 4.7, 21.8] vs. 19.6 h [IQR 6.2, 27.9], *p* < 0.001) and coronary revascularisation (median 8.2 h [IQR 4.9, 24.0] vs. 22.0 h [IQR 6.3, 46.1], *p* < 0.001) were both significantly shorter in patients with NSTEMI-OMI versus NSTEMI-NOMI. Five-year mortality was comparable in patients with NSTEMI-OMI versus NSTEMI-NOMI (adjHR 1.13 [95% CI 0.78–1.63], *p* = 0.51).

**Conclusion:**

One in three patients with type I NSTEMI had NSTEMI-OMI. These patients more often present with very high-risk clinical, ECG, and biomarker features and receive earlier invasive management. Likely related to the latter, 5-year mortality was similar between NSTEMI-OMI and NSTEMI-NOMI.

**Graphical Abstract:**

**Figure:**
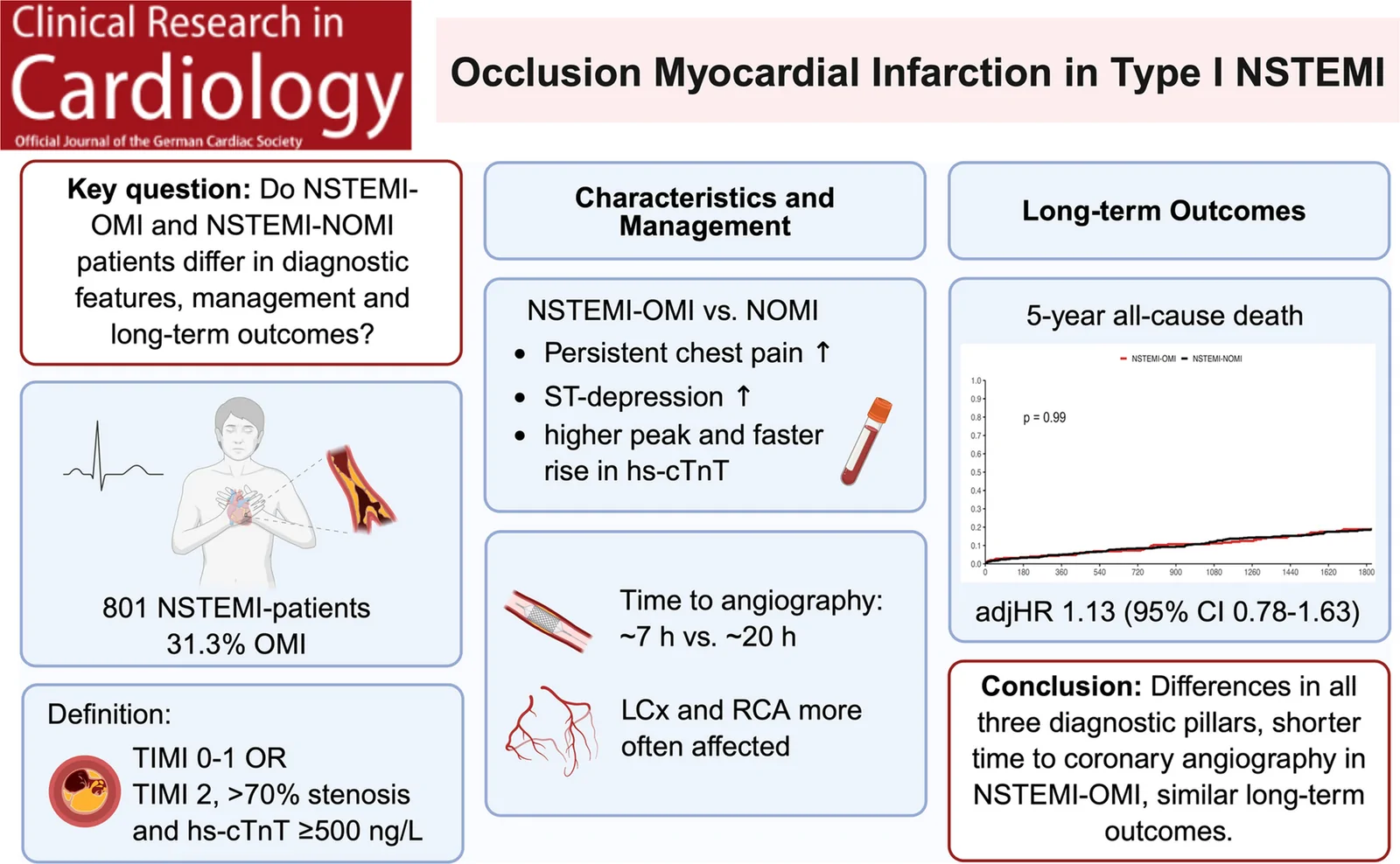

**Supplementary Information:**

The online version contains supplementary material available at https://doi.org/10.1007/s00392-026-02966-8.

## Introduction

Despite major advances in the management of acute myocardial infarction (AMI) in the past decades, long-term mortality and morbidity remain high [1]. This may, in part, be due to a disconnect between current clinical phenotypes of AMI according to the presence or absence of ST-segment elevation in the 12-lead ECG and the angiographic phenotype according to the presence or absence of complete occlusion of an epicardial segment of the culprit coronary artery [2, 3]. ST-segment elevation myocardial infarction (STEMI), which can be identified rapidly by the 12-lead ECG, is typically associated with complete coronary artery occlusion. However, a substantial proportion of patients with non-ST-segment elevation myocardial infarction (NSTEMI), often without overt ECG changes, may also present with total coronary artery occlusion [4–7]. This has led to proposals to change the paradigm, emphasising an occlusion myocardial infarction (OMI) approach rather than focusing solely on ST-segment elevation criteria [8, 9]. It has been hypothesised that NSTEMI patients with a complete coronary occlusion (NSTEMI-OMI) experience larger infarcts and worse long-term outcomes than NSTEMI patients without total coronary occlusion (NSTEMI-NOMI) and may derive benefit from very early revascularisation [3, 6, 10–12]. However, prior studies supporting the concept of NSTEMI-OMI had important methodological limitations, and major uncertainties remain regarding these assumptions. Also, the non-invasive detection of NSTEMI-OMI remains a major unmet clinical need.

To address these major gaps in knowledge, we performed a secondary analysis of a large, prospective, international multicentre diagnostic study with extensive characterisation including time since chest pain onset and central adjudication of patients presenting with suspected AMI to the emergency department (ED). Our study aimed to evaluate the prevalence of NSTEMI-OMI among consecutive type I NSTEMI patients and identify clinical, laboratory, and ECG characteristics, as well as long-term outcome in patients with NSTEMI-OMI.

## Methods

### Study design and population

**Fig. 1:**
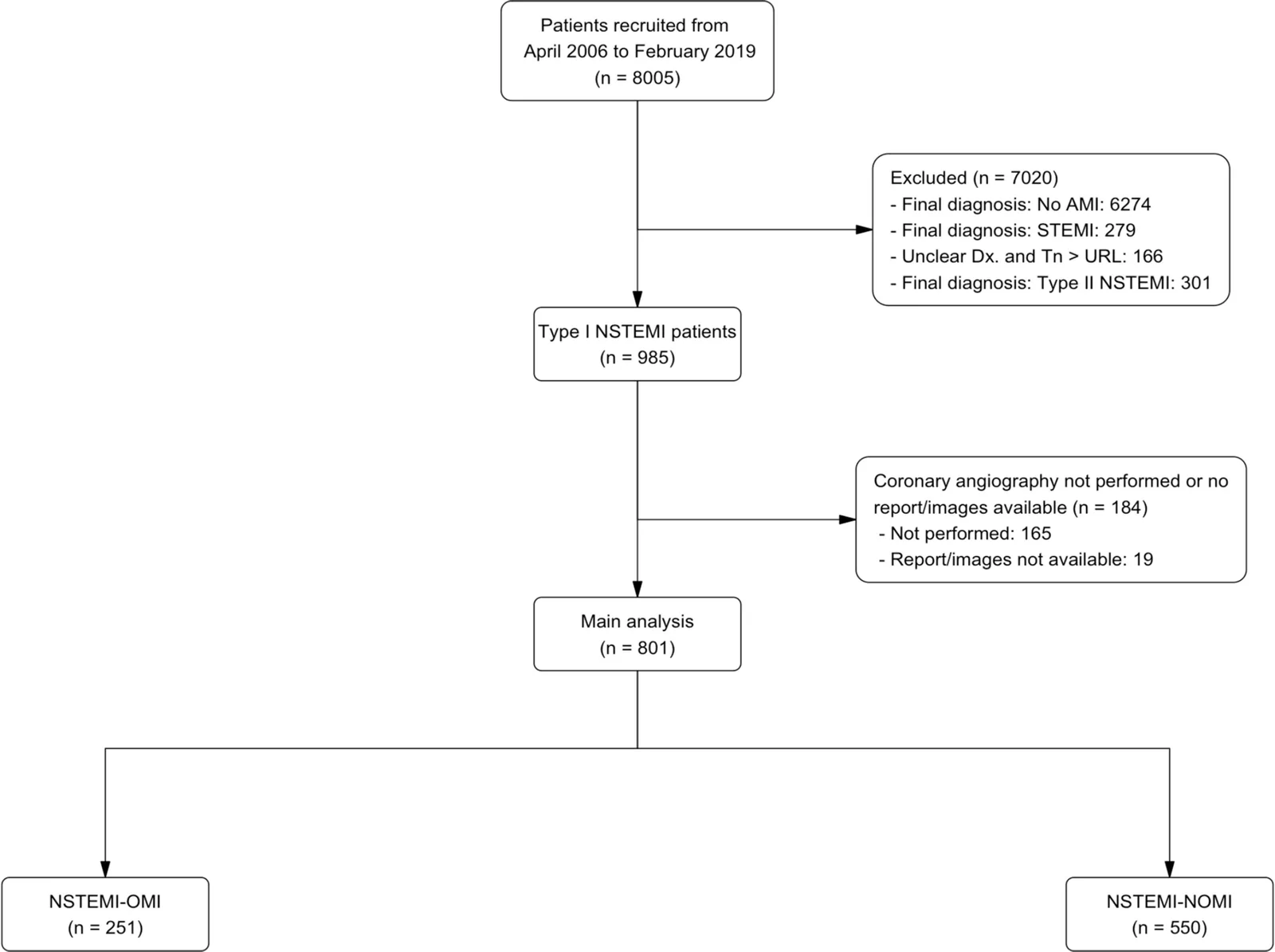
Patient flowchart. Patients were classified as NSTEMI-OMI if they had a thrombolysis in myocardial infarction (TIMI) flow grade of 0–1 or a TIMI flow grade of 2 with severe coronary narrowing (> 70%) and a peak hs-cTnT ≥ 500 ng/L. STEMI: ST-segment elevation myocardial infarction; NSTEMI: non-ST-segment elevation myocardial infarction; AMI: acute myocardial infarction; OMI: occlusion myocardial infarction; NOMI: non-occlusion myocardial infarction; Dx.: final diagnosis; Tn: clinical troponin T or I; URL: upper reference limit

This study was a secondary analysis from the Advantageous Predictors of Acute Coronary Syndromes Evaluation (APACE) study, a prospective, multicentre, international diagnostic study (NCT00470587) [13–17], conducted in 12 emergency departments (ED) in five European countries. Adult patients presenting to the ED with acute chest discomfort suggestive of AMI were enrolled from April 2006 to February 2019. All patients received prospective standardised assessment, including 34 predefined and prospectively recorded chest pain characteristics including time since chest pain onset and pain intensities measured on a visual analogue scale (VAS), ischaemic ECG findings, and serial high-sensitivity cardiac troponin T (hs-cTnT measurements). For this secondary analysis, patients were included if they had an adjudicated final diagnosis of type I (including also type 4b or 4c) NSTEMI, received coronary angiography, and had detailed coronary angiography loops and/or reports (Fig. 1). STEMI patients, including those with posterior STEMI shown in the ECG with ST-segment elevation in V7–V9, type II NSTEMI patients, patients without AMI, and patients with an unknown final diagnosis were excluded from the analysis. The study was carried out according to the principles of the Declaration of Helsinki and approved by the local ethics committees. Written informed consent was obtained from all patients.

### Central adjudication

Adjudication of the final diagnosis was performed centrally at the core laboratory of the University Hospital of Basel, Switzerland, by two independent cardiologists in line with current guidelines and the Fourth Universal Definition of Myocardial Infarction [3, 18]. In situations of disagreement, a third cardiologist was involved. The diagnostic procedures, routine clinical assessment, and the adjudication process have been described in detail previously [13, 14, 19].

### Blood sampling and laboratory methods

Blood samples for determination of Elecsys hs-cTnT (Roche Diagnostics, Rotkreuz, Switzerland) were measured as part of routine clinical care and from study blood samples. The 99th percentile has been reported at a concentration of 14 ng/L with a CV of < 10% and a limit of detection at 5 ng/L, consistent with the assay description. Additional biomarkers used in this analysis and measured at ED presentation were N-terminal pro B-type natriuretic peptide (NT-proBNP), high-sensitivity C-reactive protein (hs-CRP), interleukin-6 (IL6), and cardiac myosin binding protein C3 (cMyC). Details of the cMyC assay used have been described previously [20] and are provided in the supplemental material. The estimated glomerular filtration rate (GFR) was determined using the chronic kidney disease epidemiology collaboration formula.

### TIMI flow assessment

Thrombolysis in myocardial infarction (TIMI) flow grades were assessed from coronary angiography reports and images by two independent experienced interventional cardiologists and classified in line with the standard grading system, with 0 for no perfusion, 1 for penetration without perfusion, 2 for partial perfusion, and 3 for complete perfusion [18]. Peak troponin was defined as the highest value of hs-cTnT measured during the index case and up to 5 days from ED presentation.

### OMI definition

For our primary analysis, we used the pre-defined OMI definition proposed in the pivotal study by Al-Zaiti et al. [21], which defined OMI as angiographic evidence of an acute culprit lesion with a TIMI flow grade of 0–1 or a TIMI flow grade of 2 in the presence of severe coronary stenosis (> 70%) and a peak hs-cTnT ≥ 500 ng/L (upper reference limit [URL] 14 ng/L). All type I NSTEMI patients not meeting these criteria were labelled as NSTEMI-NOMI.

However, more recently, several alternative OMI definitions have been proposed by other investigators [9, 11, 12, 22]. To assess the robustness of our findings, we additionally performed two sensitivity analyses using these alternative definitions: (1) a more conservative definition restricted to TIMI flow grades 0–1 and (2) a more liberal definition including TIMI flow grades 0–1 or TIMI flow grades 2–3 in combination with peak hs-cTnT ≥ 1000 ng/L, as previously described by other groups [9, 11, 12, 22, 23].

### Clinical endpoints

The diagnostic endpoint was the detection of OMI at the ED. The prognostic endpoint was 5-year all-cause mortality. Secondary prognostic endpoints were major adverse cardiac events (MACE), defined as cardiovascular (CV) death, recurrent myocardial infarction (MI), or rehospitalisation due to heart failure during the follow-up as well as solely CV death or recurrent MI.

### Risk scores

The Global Registry of Acute Coronary Events Risk predictor (GRACE) and the History, ECG, Age, Risk factors, and Troponin (HEART) risk scores were calculated as previously reported [24].

### Patient follow-up

After hospital discharge, patients were contacted after 3, 12, 24, and 60 months by telephone calls or in written form. Information regarding death was furthermore obtained from the national registry on mortality, the hospital’s diagnosis registry, and the family physician’s records.

### Statistical analysis

Categorical variables were reported as counts and frequencies, continuous variables as medians with interquartile ranges (IQR). Differences in baseline characteristics between groups were assessed using the Mann–Whitney test for continuous variables and the Pearson chi-squared test for categorical variables. 95% confidence intervals (CI) were calculated using Wilson’s method if not specified otherwise. Positive likelihood ratios (pLRs) with 95% CIs were calculated to assess the value of each chest pain characteristic and ECG finding for the diagnosis of an OMI in type I NSTEMI patients. To assess which diagnostic findings at the ED were associated with earlier coronary angiography, an ordinal least squares regression model was fitted, and difference in time to coronary angiography was visualised using partial conditional effect plots. The variables included in the model were selected based on previous literature and subject matter knowledge. Survival analysis was performed using log-rank test, and outcome endpoints are shown with Kaplan–Meier survival plots. Adjusted hazard ratios using Cox regression analysis are provided for between-group differences, with adjustment for age, sex, diabetes, cancer, coronary artery disease (CAD), and the GFR. The proportional hazard (PH) assumption was assessed using Schoenfeld residuals, and no violations of the assumption were detected. In both the ordinal least squares and Cox regression models, restricted cubic splines were used to model predictors with a non-linear relationship with the respective outcome of interest [25]. To evaluate association of the time from presentation to coronary angiography and revascularisation and long-term mortality in patients with NSTEMI-OMI, we developed a dose–response model adjusted for age, sex, diabetes, cancer, CAD, and GFR for all patients with the time of coronary angiography available (*n* = 680). Patients with missing time stamps were excluded from this secondary analysis. NSTEMI-OMI patients who underwent coronary angiography either before or after a reference time of 12 h were then compared [26, 27] and their respective hazard ratios calculated.

Statistical significance was set at *p* < 0.05. No formal adjustment for multiple testing was performed; therefore, *p* values should be interpreted with caution. Data were analysed independently by two researchers using statistical software R, version 4.3.3 (R Foundation for Statistical Computing, Vienna, Austria).

## Results

### Type I NSTEMI: baseline characteristics and OMI prevalence

Among 985 patients with an adjudicated type I NSTEMI, 165 were excluded because coronary angiography was not performed and 19 patients were excluded due to missing interventional reports or images (Fig. 1). A total of 801 patients with adjudicated type 1 NSTEMI were eligible for the main analysis (median age 68 years, 22.2% female), of which 251 patients (31.3%) were adjudicated as NSTEMI-OMI (Table 1).

**Table 1:** Baseline characteristics

Patient characteristics	NSTEMI-OMI (*n* = 251)^a^	NSTEMI-NOMI (*n* = 550)^a^	*p* value^b^
Demographics			
Age	68.0 (57.0, 77.0)	69.0 (58.0, 77.0)	0.5
Female	36 (14.3%)	142 (25.8%)	< 0.001
Cardiovascular risk factors			
Arterial hypertension	178 (70.9%)	414 (75.3%)	0.2
Dyslipidaemia	148 (59.0%)	366 (66.5%)	0.038
Obesity (BMI > 30 kg/m^2^)	55 (22.0%)	133 (24.4%)	0.5
Diabetes	62 (24.7%)	140 (25.5%)	0.8
Smoking history	176 (70.1%)	377 (68.9%)	0.7
Medical history			
Coronary artery disease	96 (38.2%)	236 (42.9%)	0.2
Myocardial infarction	79 (31.5%)	176 (32.0%)	0.9
PCI	66 (26.3%)	185 (33.6%)	0.038
CABG	37 (14.7%)	71 (12.9%)	0.5
PAD	27 (10.8%)	47 (8.5%)	0.3
Stroke	21 (8.4%)	39 (7.1%)	0.5
Pulmonary embolism	8 (3.2%)	16 (2.9%)	0.8
Chronic medication			
Antiplatelet drug	109 (43.4%)	282 (51.3%)	0.039
Anticoagulant	26 (10.4%)	38 (6.9%)	0.095
Statin	83 (33.1%)	246 (44.7%)	0.002
Baseline assessments			
Systolic BP, mmHg	142.0 (127.5, 160.5)	146.0 (130.0, 162.0)	0.14
Diastolic BP, mmHg	83.0 (74.0, 95.0)	81.0 (71.0, 92.0)	0.063
Heart rate, beats/min	75.0 (65.0, 86.0)	75.0 (65.0, 86.0)	0.9
Laboratory findings			
Haemoglobin (g/dL)	144.0 (133.0, 154.0)	143.0 (132.0, 154.0)	0.3
Thrombocytes (× 10⁹/L)	229.0 (190.0, 269.0)	225.0 (190.8, 278.0)	0.9
GFR EPI (mL/kg/1.73 m^2^)	86.0 (70.0, 99.0)	85.0 (67.0, 97.0)	0.5

*NSTEMI* non-ST-segment elevation myocardial infarction, *OMI* occlusion myocardial infarction, *NOMI* non-occlusion myocardial infarction, *PCI* percutaneous coronary intervention, *CABG* coronary artery bypass graft, *PAD* peripheral artery disease, *BP* blood pressure, *GFR* glomerular filtration rate

^a^Median (IQR); *n* (%)

^b^Wilcoxon rank sum test; Pearson’s chi-squared test

### OMI characterisation

Patients with NSTEMI-OMI were similar in age to patients with NSTEMI-NOMI (median 68 vs. 69 years), but less often female (14.3% vs. 25.8%), had less often previously diagnosed dyslipidaemia (59.0% vs. 66.5%), and had less often undergone previous PCI (26.3% vs. 33.6%, *p* = 0.038). Patients with NSTEMI-OMI were less often on chronic antiplatelet therapy (43.4% vs. 51.3%, *p* = 0.039) and statins (33.1% vs 44.7%, *p* = 0.002) but more often on oral anticoagulants (10.4% vs. 6.9%, *p* = 0.095) (Table 1).

### Chest pain characteristics and ECG findings

**Fig. 2:**
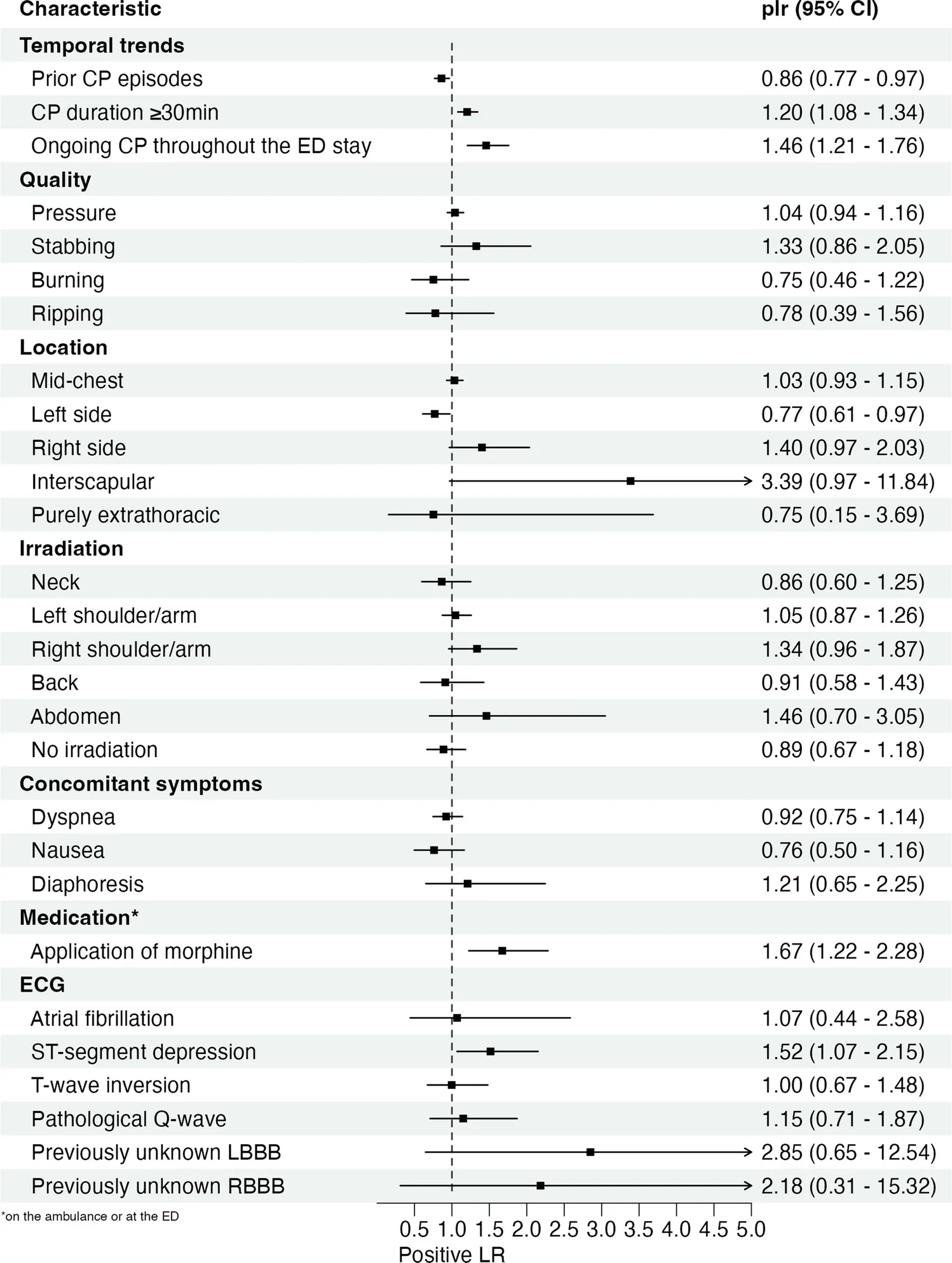
Positive likelihood ratio for chest pain and ECG characteristics at presentation. Positive likelihood ratios (LRs) (and their 95% CIs) of predefined chest pain (CP) characteristics and findings on electrocardiography (ECG) for the classification as an occlusion myocardial infarction (OMI) in type I non-ST-segment elevation myocardial infarction. ED: emergency department; LBBB: left bundle branch block; RBBB: right bundle branch block

Patients with NSTEMI-OMI versus NSTEMI-NOMI had comparable time from chest pain onset to ED presentation (median 6.3 h versus 6.0 h, *p* = 0.5) and reported similar peak pain levels (both VAS 7 [IQR 5, 8], *p* = 0.2). Patients with NSTEMI-OMI had less often experienced previous chest pain episodes (60.9% vs. 70.7%, *p* = 0.006), the pain episode leading to presentation lasted more commonly longer than 30 min (70.5% vs. 59.0%, *p* = 0.002), were more often treated with morphine (24.3% vs. 16.4%, *p* = 0.008), and more often had persistent chest pain at the time of transfer from the ED compared to patients with NSTEMI-NOMI (56.1% vs. 37.9%, *p* < 0.001) (Supplemental Table 1 and Fig. 2). Patients with NSTEMI-OMI presented more often with ST-segment depression in the 12-lead ECG at ED presentation (34.3% vs. 23.0%, *p* < 0.001) and with a not previously known left bundle branch block (LBBB) (3.2% vs. 0.9%, *p* = 0.030) (Supplemental Table 2).

**Table 2:** Biochemical characterisation

Laboratory marker	*n*	NSTEMI-OMI (*n* = 251)^a^	NSTEMI-NOMI (*n* = 550)^a^	*p* value^b^
Cardiomyocyte injury				
0 h hs-cTnT (ng/L)	791	131 (39, 330)	52 (26, 107)	< 0.001
1 h hs-cTnT (ng/L)	635	188 (56, 411)	66 (34, 139)	< 0.001
Delta 0/1 h hs-cTnT (ng/L)	633	15 (4, 56)	7 (1, 26)	< 0.001
Peak hs-cTnT (ng/L)	801	1220 (493, 2631)	157 (55, 387)	< 0.001
Myosin binding protein C3	494	857 (241, 2395)	165 (71, 428)	< 0.001
Haemodynamic cardiac stress				
NT-proBNP (ng/L)	494	562 (143, 1630)	328 (112, 874)	0.006
Inflammation				
Leukocytes (× 10⁹/L)	793	8.93 (7.32, 11.00)	7.83 (6.50, 9.70)	< 0.001
hs-CRP (mg/L)	483	3 (1, 7)	2 (1, 6)	0.013
IL-6 (ng/L)	494	6 (3, 10)	4 (2, 7)	< 0.001

*NSTEMI* non-ST-segment elevation myocardial infarction, *OMI* occlusion myocardial infarction, *NOMI* non-occlusion myocardial infarction, *h* hour, *hs-cTnT* high-sensitivity cardiac troponin T, *cMyc* cardiac myosin binding protein C3, *NT-proBNP* N-terminal pro B-type natriuretic peptide, *hs-CRP* high-sensitivity C-reactive protein, *IL-6* interleukin 6

^a^Median (IQR)

^b^Wilcoxon rank sum test

### Laboratory findings

NSTEMI-OMI patients showed significantly higher levels of 0 h hs-cTnT (131 ng/L [IQR 39, 330] vs. 52 ng/L [IQR 26, 107], *p* < 0.001), 1 h hs-cTnT (188 ng/L [IQR 56, 411] vs. 66 ng/L [IQR 34, 139], *p* < 0.001), a greater 0 h to 1 h delta of hs-cTnT (15 ng/L [IQR 4, 56] vs. 7 ng/L [IQR 1, 26], *p* < 0.001), higher levels of 0 h cMyC (857 ng/L [IQR 241, 2395] vs. 165 ng/L [71, 428]), and higher levels of NT-proBNP (562 ng/L [IQR 143, 1630] vs. 328 ng/L [IQR 112, 874], *p* = 0.006) at the time of ED presentation. Median peak hs-cTnT within 5 days was 1220 ng/L (IQR 493, 2631) in NSTEMI-OMI and 157 ng/L (IQR 55, 387) in NSTEMI-NOMI patients (*p* < 0.001, Table 2).

### Risk scores

Patients with NSTEMI-OMI had comparable GRACE and HEART score to patients with NSTEMI-NOMI (GRACE score 109 [84–128] vs. 106 [82–125] and HEART score 7 [6–8] vs. 7 [6–8], respectively). When stratifying in low, intermediate, and high risk, results were also comparable for the GRACE score (low risk 29.1% vs. 30.1%; moderate risk 31.6% vs. 35.1%; high risk 39.3% vs. 34.8%, respectively) and HEART score (low risk 1.2% vs. 1.7%; moderate risk 34.8% vs. 39.8%; high risk 64% vs. 58.6%, respectively).

### Angiographic findings

A total of 162 (64.5%) of the NSTEMI-OMI patients were classified with an acute vessel occlusion and TIMI flow grade 0 or 1, whilst 89 patients (35.5%) were labelled as NSTEMI-OMI with TIMI flow grade 2, a coronary narrowing of > 70%, and a peak hs-cTnT ≥ 500 ng/L (Supplemental Table 3). The distribution of culprit arteries differed between patients with NSTEMI-OMI versus NSTEMI-NOMI. In NSTEMI-OMI, the left circumflex artery (LCx, 31.4%), the left anterior descending artery (LAD, 27.7%), and the right coronary artery (RCA, 27.3%) each accounted for roughly one-third of culprit lesions. In contrast, in NSTEMI-NOMI, the LAD was more frequently the culprit (38.7%), compared to the LCx (17.8%) and RCA (19.0%) (*p* < 0.001).

**Table 3:** Patient disposition and management

	NSTEMI-OMI (*n* = 251)^a^	NSTEMI-NOMI (*n* = 550)^a^	*p* value^b^
Patient disposition			
Time at the ED (h)	2.9 (2.0, 5.3)	4.3 (2.5, 7.2)	< 0.001
Hospitalisation	238 (95%)	518 (94%)	0.7
Time from ED presentation to coronary angiography (h)	7.2 (4.7, 21.8)	19.6 (6.2, 27.9)	< 0.001
Interventions			
Revascularisation performed	231 (92%)	463 (84%)	0.002
Time from ED presentation to revascularisation (h)^*^	8.2 (4.9, 24.0)	22.0 (6.3, 46.1)	< 0.001
Revascularisation type			0.7
Balloon dilatation	6 (2.6%)	16 (3.5%)	
Stent implantation	198 (86%)	385 (83%)	
CABG	27 (12%)	62 (13%)	
Medication at discharge			
Antiplatelet drug	244 (97%)	533 (97%)	0.8
Anticoagulant	43 (17%)	59 (11%)	0.012
Statin	238 (95%)	504 (92%)	0.11
Beta-blocker	210 (84%)	442 (80%)	0.3
Diuretic drug	75 (30%)	188 (34%)	0.2
ACE-I/ARB	215 (86%)	444 (81%)	0.09
Ca-antagonist	43 (17%)	108 (20%)	0.4
Nitrates	41 (16%)	112 (20%)	0.2

*NSTEMI* non-ST-segment elevation myocardial infarction, *OMI* occlusion myocardial infarction, *NOMI* non-occlusion myocardial infarction, *CPO* chest pain onset, *ED* emergency department, *h* hours, *CABG* coronary artery bypass graft, *ACE-I* angiotensin-converting enzyme inhibitor, *ARB* angiotensin II receptor blocker

^*^Time to revascularisation was defined as time from ED presentation to coronary angiography for patients who received percutaneous coronary intervention and time from ED presentation to surgery for patients who underwent CABG surgery

^a^Median (IQR); *n* (%)

^b^Wilcoxon rank sum test; Pearson’s chi-squared test; Fisher’s exact test

### Management

Time from ED presentation to coronary angiography and invasive revascularisation were significantly shorter in patients with NSTEMI-OMI than in patients with NSTEMI-NOMI (median 7.2 h [IQR 4.7, 21.8] vs. 19.6 h [IQR 6.2, 27.9], and median 8.2 h [IQR 4.9, 24.0] vs. 22.0 h [IQR 6.3, 46.1], both *p* < 0.001) (Table 3). Among clinical characteristics, the 12-lead ECG and hs-cTnT, the variables associated with shorter time to coronary angiography were younger age, male sex, ST-depression in the 12-lead ECG, application of morphine, and a higher hs-cTnT concentration (Supplemental Fig. 1 and Supplemental Table 4).

### Long-term outcomes

Through 5 years, 143 deaths (17.9%) occurred, of which 90 were CV deaths (11.2%). A recurrent MI occurred in 108 patients (13.4%), and 52 patients (6.5%) were hospitalised for heart failure.

**Fig. 3:**
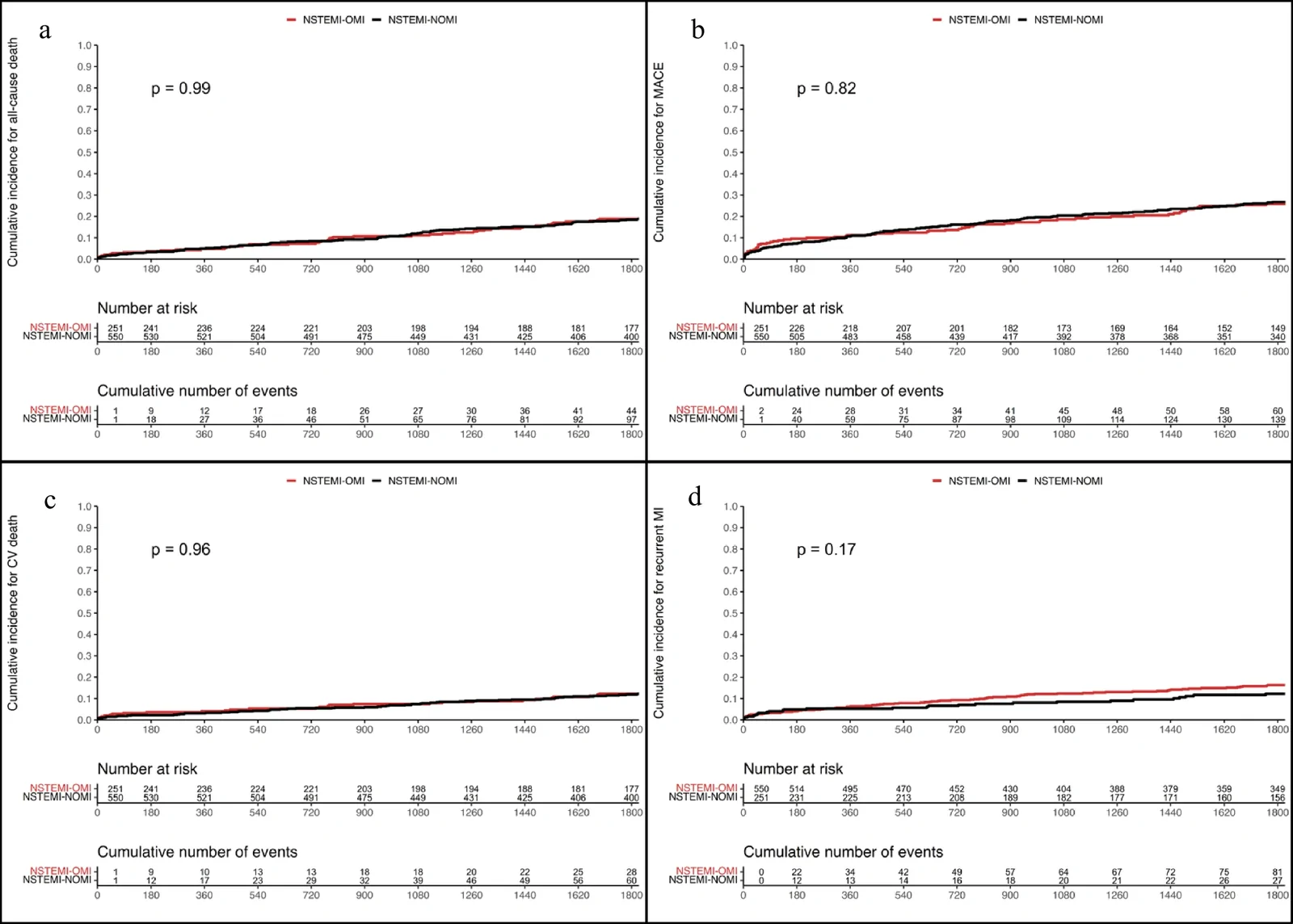
Kaplan–Meier curves for (**a**) all-cause death, (**b)** MACE, (**c)** CV death, and (**d**) recurrent MI over 5 years. Cumulative incidents for different endpoints over 5 years. The *x*-axis depicts the follow-up time in days. MACE is defined as cardiovascular death, recurrent myocardial infarction, or rehospitalisation due to heart failure within 5 years. NSTEMI: non-ST-segment elevation myocardial infarction; OMI: occlusion myocardial infarction; NOMI: non-occlusion myocardial infarction; MACE: major adverse cardiac event; CV: cardiovascular; MI: myocardial infarction

The cumulative incidence of all-cause death, MACE, CV death, or recurrent MI over 5 years was comparable between patients with NSTEMI-OMI and NSTEMI-NOMI (Fig. 3). In a multivariable Cox model, the adjusted hazard ratio for all-cause death for NSTEMI-OMI was 1.13 (95% CI 0.78–1.63, *p* = 0.51) and 1.04 (95% CI 0.76–1.43, *p* = 0.79) for MACE.

### Sensitivity analyses

Sensitivity analyses showed that the prevalence of NSTEMI-OMI was susceptible to the specific definition used, ranging from 20.3% to 32.1%. Patients with NSTEMI-OMI according to the more liberal OMI definition had a higher 5-year mortality risk (adjHR 1.43 [95% CI 1.004–2.04, *p* = 0.05]). All other prognostic findings were consistent throughout different definitions of NSTEMI-OMI (Table 4).

**Table 4:** Adjusted hazard ratios for 5-year outcomes for different definitions of NSTEMI-OMI

NSTEMI-OMI definition	NSTEMI-OMI, *n* (%)	All-cause death, adjHR (95% CI)	MACE, adjHR (95% CI)	CV death, adjHR (95% CI)	Recurrent MI, adjHR (95% CI)
**Main analysis**					
TIMI flow grade 0–1 OR TIMI flow grade 2 with severe coronary narrowing (> 70%) and hs-cTnT ≥ 500 ng/L	251 (31.3)	1.13 (0.78–1.63), *p* = 0.51	1.04 (0.76–1.43), *p* = 0.79	1.14 (0.72–1.81), *p* = 0.58	0.73 (0.47–1.14), *p* = 0.17
**Sensitivity analyses**					
TIMI flow grade 0–1	163 (20.3)	1.29 (0.85–2), *p* = 0.23	1.1 (0.77–1.58), *p* = 0.61	1.22 (0.71–2.08), *p* = 0.46	0.78 (0.45–1.32), *p* = 0.35
TIMI flow grade 0–1 OR TIMI flow grade 2–3 with hs-cTnT ≥ 1000 ng/L	257 (32.1)	1.43 (1.004–2.04), *p* = 0.05	1.08 (0.8–1.47), *p* = 0.6	1.33 (0.85–2.06), *p* = 0.21	0.70 (0.45–1.1), *p* = 0.12

Adjusted hazard ratios for primary and secondary outcome, when patients are stratified according to different definitions for NSTEMI-OMI. Multivariable Cox proportional hazards models were adjusted for age, sex, GFR, diabetes mellitus, history of CAD, and history of malignancy. MACE is defined as cardiovascular death, recurrent myocardial infarction, or rehospitalisation due to heart failure during follow up

*NSTEMI* non-ST-segment elevation myocardial infarction, *OMI* occlusion myocardial infarction, *MACE* major adverse cardiac event, *TIMI* thrombolysis in myocardial infarction, *adjHR* adjusted hazard ratio, *CI* confidence interval, *CV* cardiovascular, *MI* myocardial infarction, *hs-cTnT* high-sensitivity cardiac troponin T, *GFR* glomerular filtration rate, *CAD* coronary artery disease

### Association between time to coronary angiography and mortality in NSTEMI-OMI

**Fig. 4:**
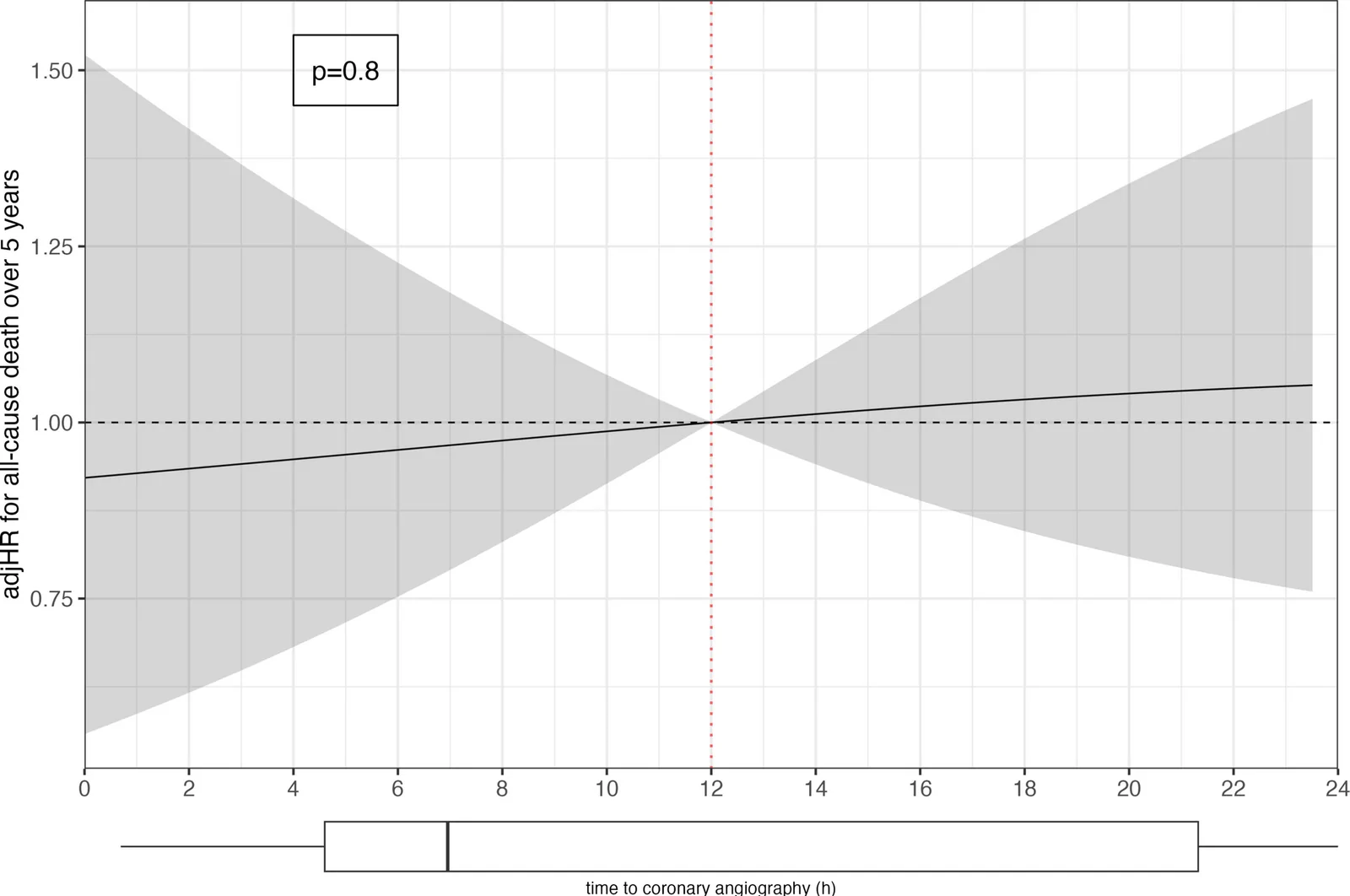
Association of time to coronary angiography and 5-year all-cause death. Association between time from presentation to coronary angiography (in hours) and the adjusted hazard ratio (adjHR) for all-cause death over 5 years for type I non-ST-segment elevation myocardial infarction NSTEMI patients with an occlusion myocardial infarction (OMI). The underlying Cox proportional hazards model was adjusted for age, sex, GFR, diabetes mellitus, history of CAD, and history of malignancy. The solid line represents the adjusted hazard ratio, and the shaded area indicates the 95% confidence interval (CI). The dashed line at an adjHR of 1.0 serves as the reference, denoting no association. Time is modelled continuously, and the adjHR is calculated relative to the reference point of 12 h time to coronary angiography. The box plot shows the patient distribution regarding time to coronary angiography (median 7.1 h, interquartile range 4.7–22 h)

Within the NSTEMI-OMI group with time stamps for coronary angiography available, 41 events occurred. There was absence of evidence of an association between time to coronary angiography and mortality (*p* = 0.803) (Fig. 4) or the secondary endpoints (Supplemental Figs. 2 and 3).

## Discussion

This secondary analysis of a large, international, multicentre study was performed to evaluate the prevalence, characteristics, management, and outcome of NSTEMI-OMI. We report seven major findings:

First, about one in three (31.3%) patients presenting with type I NSTEMI had NSTEMI-OMI. This extends and corroborates the results of prior retrospective studies and a systematic review and meta-analysis, reporting prevalences of 25.5 to 34% [28–30]. Interestingly, sensitivity analyses showed that the prevalence of NSTEMI-OMI was susceptible to the specific definition used, ranging from 20.3 to 32.1% for alternative previously advocated definitions. Second, NSTEMI-OMI patients differed substantially in all three diagnostic pillars of AMI and had more often very high-risk features including more often persistent chest pain, ST-segment depression in the 12-lead ECG, higher hs-cTnT and cMyC concentrations at ED presentation, and higher 0 h to 1 h hs-cTnT increases as well as higher peak hs-cTnT concentrations. This indicates that many patients with NSTEMI-OMI may be identified as ‘very high-risk NSTEMIs’ already in the ED using established clinical diagnostic tools. Importantly, established risk scores such as GRACE and HEART score did not differ between NSTEMI-OMI and NSTEMI-NOMI patients. This may, in part, be explained by the fact that hs-cTn, the strongest predictive variable, is incorporated as a binary rather than a continuous variable based on the upper reference limit, potentially resulting in a loss of predictive information. Third, the distribution of culprit coronary arteries differed between patients with NSTEMI-OMI and NSTEMI-NOMI. Whilst in NSTEMI-OMI the LCx was the most affected, in NSTEMI-NOMI the LAD was most often the culprit. This observation is in line with similar findings from meta-analyses [4, 5]. Fourth, to the best of our knowledge, this is the first study to show that, likely as a consequence of appropriate detection as ‘very high-risk NSTEMIs’ in the ED, the time from presentation to coronary angiography (median 7.2 h versus 19.6 h) and coronary revascularisation (median 8.2 h versus 22.0 h) was significantly shorter in patients with NSTEMI-OMI compared to NSTEMI-NOMI. Therefore, management in this contemporary cohort differs markedly from that applied in the studies included in previous meta-analyses which were conducted prior to the adoption of early coronary angiography strategies in patients with very high-risk NSTEMI in current clinical guidelines [3, 31]. In these studies, mean time to angiography or PCI was 31.3 h in patients with NSTEMI-OMI and 34.2 h in NSTEMI-NOMI, respectively [4, 5]. Fifth, the variables that were associated with a shorter time to coronary angiography were younger age, male sex, ST-depression in the 12-lead ECG, application of morphine, and higher hs-cTnT. Sixth, patients with NSTEMI-OMI versus NSTEMI-NOMI had comparable 5-year all-cause mortality, CV mortality, and MACE rates. Sensitivity analyses confirmed this observation across different definitions of NSTEMI-OMI. This finding contrasts with previous studies that showed higher rates of death and MACE in patients with NSTEMI-OMI as compared to NSTEMI-NOMI [4–6, 11, 28]. One possible explanation is that timely initiation of appropriate treatments, including early coronary revascularisation in patients with ‘very high-risk NSTEMI’, many of them with NSTEMI-OMI, in this current cohort might have mitigated the excess mortality observed in the earlier studies [4–6, 11, 28]. This hypothesis is supported by data from large cohorts of patients with STEMI and meta-analysis of randomised controlled trials showing benefits of early versus delayed revascularisation in patients with very high-risk NSTEMI [3, 31, 32]. Although the observed association is compatible with a mitigation in events by earlier invasive strategy, residual confounding and selection bias may remain and thus causal interpretation should be avoided. Seventh, there was no association between time to coronary angiography and long-term mortality in patients with NSTEMI-OMI, indicating similar long-term outcomes in patients receiving very early (e.g. < 12 h) invasive treatment compared to within 12–24 h. However, this secondary analysis may have been underpowered. Interpretation of these results should merely be hypothesis generating.

These findings extend and corroborate prior research aiming to advance the diagnosis, characterisation, and management of patients with NSTEMI [3–6, 8, 11, 28, 31, 32]. They also have important clinical implications. First, awareness of and attention to guideline-recommended ‘very high-risk’ NSTEMI features including persistent chest pain, ST-segment depression, very high initial hs-cTnT concentrations, and very high increase in hs-cTnT concentration from 0 to 1 h are mandated. These features help facilitate both a risk-adjusted and benefit-adjusted timing of coronary revascularisation. Second, many patients with NSTEMI-OMI can be identified as ‘very high-risk NSTEMIs’ already in the ED using established clinical diagnostic tools, such as hs-cTnT, to be triaged to rapid coronary revascularisation. Indeed, NSTEMI-OMI patients had a shorter time from admission to coronary angiography in comparison to NSTEMI-NOMI patients. Whilst this may question the need for recently developed machine learning–based solutions aiming to help in the diagnosis of NSTEMI-OMI [21, 22], two important considerations must be taken into account. First, the median time to coronary admission was 7.2 h, which is not aligned with STEMI-like care. Machine learning–based models held the promise of detecting faster NSTEMI-OMI and thus aid to reduce time to coronary admission and continue to improve outcomes. This should be tested in implementation studies. Second, established risk scores such as the GRACE and HEART score did not differ between NSTEMI-OMI and NSTEMI-NOMI and should thus not be used for suspecting OMI among NSTEMI patients.

It is important to highlight that many open questions remain, including whether STEMI-like care including very early coronary angiography and revascularisation may provide further benefit in NSTEMI-OMI. Although this study did not find an association between the time to coronary angiography and revascularisation in patients with NSTEMI-OMI, the study was underpowered for this secondary analysis as the number of events was low (*n* = 78), resulting in wide confidence intervals. Future large appropriately powered randomised controlled trials will be needed to provide clear answers. Similar uncertainties remain regarding the timing of the initiation of dual antiplatelet therapy [3, 31]. Additional open questions include the possible clinical utility of novel cardiovascular biomarkers including cMyC for the earlier detection of NSTEMI-OMI. The observed very pronounced difference of cMyC concentrations at ED presentation among NSTEMI-OMI vs. NSTEMI-NOMI patients hints to a possible clinical utility and is supported by both pathophysiological differences including faster release kinetics and higher abundance compared to hs-cTn and higher diagnostic accuracy in early presenters [20, 33–36].

Several limitations need to be acknowledged. First, despite the robust sample size, the study may have limited power to detect mortality differences across subgroups. Second, the observational design of the study does not allow to prove causality. Third, unmeasured confounding variables may have influenced our findings. Fourth, there is no universally accepted definition of OMI, which limits the comparability between studies. However, we performed a sensitivity analysis using various OMI definitions, all of which showed consistent results regarding the prognostic findings, thus enhancing the external validity. Fifth, as most OMI definitions include hs-cTn thresholds, which are inherently subjective, there is a potential risk of misclassification. However, OMI definitions relying exclusively on angiographic findings (e.g. TIMI flow grades 0–1) are also susceptible to misclassification. Specifically, spontaneous coronary reperfusion may result in false-negative classification, whilst uncertainty in distinguishing acute from chronic total occlusions may lead to false-positive classification in some patients. Hopefully, a consensus on a uniform definition on OMI will emerge in the near future. Sixth, whilst clinical characteristics, ECG features, biomarker values, and coronary angiography findings were reported in detail in this analysis, information regarding the timing of echocardiography and the pharmacological treatments administered was unavailable for most patients. Consequently, it remains unclear whether the use of echocardiography may have contributed to shorter times to coronary angiography. Future studies should specifically address this important question. Finally, in clinical practice, not all patients with type I NSTEMI undergo coronary angiography, e.g. frail patients with extensive comorbidities, particularly if asymptomatic at the time of ED presentation, and may be treated conservatively. Due to the necessity of coronary angiography findings for the classification as NSTEMI-OMI or NSTEMI-NOMI, the prevalence estimates presented here apply to an invasively managed, angiographically available type I NSTEMI subset. Thus, the incidence, characteristics, and outcomes of patients with NSTEMI-OMI not undergoing coronary angiography were not investigated in this study and remain unknown.

## Conclusion

One in three patients with type I NSTEMI presents with NSTEMI-OMI, involving significantly more often the LCx and RCA as in NSTEMI-NOMI. Patients with NSTEMI-OMI often exhibit guideline-recommended ‘very high-risk’ features, including persistent chest pain, ST-segment depression, and very high rise in hs-cTnT, and accordingly had much shorter time to coronary angiography and revascularisation compared with NSTEMI-NOMI. Likely related to earlier treatment in patients with NSTEMI-OMI, long-term mortality and MACE rates were similar between groups.

## Supplementary Information

Below is the link to the electronic supplementary material.

Supplementary Material 1 (DOCX 1.67 MB)

## Data Availability

The data underlying this article will be shared on reasonable request to the corresponding authors.
