# Supplementary material for: Occlusion myocardial infarction in type I NSTEMI: prevalence, characteristics, management, and long-term outcome
Source: Clin Res Cardiol. 2026 Jul 22;115(10):1870–83. doi: 10.1007/s00392-026-02966-8 (PMC13562278; doi:10.1007/s00392-026-02966-8)
Supplement: Supplementary file 1 [file 392_2026_2966_MOESM1_ESM.docx]

**Occlusion myocardial infarction in type I NSTEMI: Prevalence, characteristics, management, and long-term outcome**

**Supplemental Material**

Lea Kirsten^1*^, MD; Pedro Lopez-Ayala, MD^1*^; Emel Kaplan, MD PhD^1^; Jasper Boeddinghaus, MD^1^; Luca Koechlin, MD^1,2^; Lourdes Herraiz-Recuenco, MSc^1^; Gabrielle Huré, MSc^1^; Karin Wildi, MD PhD^3^; Paolo Bima, MD^1,4^; Jonas Glaeser, MD^1^; Carlos C. Spagnuolo, MD^1^; Luca Crisanti, MD^1^; Òscar Miró, MD^5^; Michael Christ, MD ^6^; Dagmar I. Keller, MD Prof.^7^; Javier F. Martin-Sanchez, MD ^8^; Roland Bingisser MD ^9^; Gregor Leibundgut MD^1^, Felix Mahfoud, MD^1^; Ivo Strebel, PhD^1^; Christian Mueller, MD^1^ on behalf of the APACE investigators^#^

^1^Cardiovascular Research Institute Basel (CRIB) and Department of Cardiology, University Hospital Basel, University of Basel, Basel, Switzerland; ^2^Department of Cardiac Surgery, University Hospital Zürich, University of Zürich, Switzerland; ^3^Cantonal Hospital Aarau, Department of Intensive Care; ^4^Department of Medical Sciences, University of Turin, Turin, Italy; ^5^Emergency Department, Hospital Clinic, IDIBAPS, University of Barcelona, Barcelona, Catalonia, Spain; ^6^Emergency Department, Kantonsspital Luzern, Switzerland; ^7^Emergency Department, University Hospital Zurich, Zurich, Switzerland; ^8^Servicio de Urgencias, Hospital Clínico San Carlos, Madrid, Spain; ^9^Emergency Department, University Hospital Basel, Basel, Switzerland; *Contributed equally and should be considered first author. ^#^Investigators listed at the end of the article.

***Clinical Research in Cardiology***

**Correspondance to:** [christian.mueller@usb.ch](mailto:christian.mueller@usb.ch), [pedro.lopzayala@usb.ch](mailto:pedro.lopzayala@usb.ch)

Trial Registration: **ClinicalTrials.gov number, NCT00470587**

**Supplemental Methods**

**Blood sampling and laboratory methods**

**References**

**Supplemental Tables**

**Supplemental Table 1 –** Chest pain characteristics

**Supplemental Table 2 –** ECG findings

**Supplemental Table 3 –** Angiographic findings

**Supplemental Table 4 -** Summary of the effects of each variable for time to coronary angiography

**Supplemental Figures**

**Supplemental Figure 1 -** Partial (conditional) effects plots for time to coronary angiography from ED presentation

**Supplemental Figure 2 –** Association of time to coronary angiography and secondary outcomes

**Supplemental Figure 3 –** Association of time to revascularisation and primary and secondary outcomes

**Cardiac myosing binding protein C3**

For measurements of all biomarkers, venous blood samples were collected into EDTA plasma tubes at ED presentation. After centrifugation, samples were frozen at –80 C and stored until assayed in a blinded fashion in a dedicated core laboratory. Measurement of cardiac myosin binding protein C3 (cMyC) were assayed in a blinded fashion using a robust, fully automated research prototype electrochemiluminescence immunoassay (Cobas Elecsys Immunoanalyzer e601, Roche Diagnostics). This assay functions via the sandwich principle, applying monoclonal antibodies specifically developed against cMyC (lower detection limit = 0.458 ng/L; functional sensitivity was 2.1 ng/L; patent number WO 2025/027012 A1). The Sandwich immunoassay principle and the epitopes for cMyC have been described in detail previously [1].

**References**

1.	Lopez-Ayala P, Boeddinghaus J, Koechlin L, Bima P, Glaeser J, Spagnuolo CC, et al. Incremental Value of Cardiac Myosin-Binding Protein C for the Early Diagnosis of Acute Myocardial Infarction. JACC. 2025;86(25):2616–32.

**Supplemental Tables**

**Supplemental Table 1.** Chest pain characteristics

|  | **NSTEMI-OMI**,  N = 251^1^ | **NSTEMI-NOMI**,  N = 550^1^ | **p-value**^2^ |
| --- | --- | --- | --- |
| Time since chest pain onset (h) | 6.3 (3.0, 24.0) | 6.0 (2.5, 19.9) | 0.5 |
| Previous chest pain episodes | 148 (60.9%) | 382 (70.7%) | 0.006 |
| Pain maximum (VAS) | 7.0 (5.0, 8.0) | 7.0 (5.0, 8.0) | 0.2 |
| Duration of episodes >30min | 170 (70.5%) | 315 (59.0%) | 0.002 |
| Persistent chest pain throughout the ED stay | 97 (56.1%) | 145 (37.9%) | <0.001 |
| Dyspnoea | 107 (43.9%) | 266 (48.8%) | 0.2 |
| Nausea | 46 (18.5%) | 101 (19.0%) | 0.9 |
| Diaphoresis | 19 (11.7%) | 37 (10.4%) | 0.7 |
| Application of morphine | 61 (24.3%) | 90 (16.4%) | 0.008 |
| *^1^Median (IQR); n (%)*  *^2^Wilcoxon rank sum test; Pearson's Chi-squared test* | | | |
|  | | | |

NSTEMI: Non-ST-segment elevation myocardial infarction; OMI: Occlusion myocardial infarction; NOMI: Non-occlusion myocardial infarction; VAS: Visual analogue scale; ED: Emergency department

**Supplemental Table 2.** ECG findings

|  | **NSTEMI-OMI**,  N = 251^1^ | **NSTEMI-NOMI**,  N = 550^1^ | **p-value**^2^ |
| --- | --- | --- | --- |
| Sinus rhythm | 216 (87.1%) | 497 (91.5%) | 0.052 |
| Atrial fibrillation | 20 (8.1%) | 28 (5.2%) | 0.11 |
| ST-segment depression | 85 (34.3%) | 125 (23.0%) | <0.001 |
| T-inversion | 58 (23.1%) | 150 (27.3%) | 0.2 |
| Pathological Q wave | 35 (13.9%) | 66 (12.0%) | 0.4 |
| LBBB | 13 (5.2%) | 20 (3.7%) | 0.3 |
| not know to be old | 8 (3.2%) | 5 (0.9%) | 0.030 |
| RBBB | 20 (8.1%) | 31 (5.7%) | 0.2 |
| not know to be old | 6 (2.5%) | 7 (1.3%) | 0.2 |
| *^1^n (%)*  *^2^Pearson's Chi-squared test; Fisher's exact test* | | | |
|  | | | |

NSTEMI: Non-ST-segment elevation myocardial infarction; OMI: Occlusion myocardial infarction; NOMI: Non-occlusion myocardial infarction; LBBB: Left bundle branch block; RBBB: Right bundle branch block

**Supplemental Table 3.** Angiographic findings

|  | **NSTEMI-OMI**,  N = 251^1^ | **NSTEMI-NOMI**,  N = 550^1^ | **p-value**^2^ |
| --- | --- | --- | --- |
| Diagnostic outcome |  |  | 0.007 |
| Normal Coronaries | 0 (0.0%) | 3 (0.5%) |  |
| Mild CAD | 0 (0.0%) | 13 (2.4%) |  |
| 1-vessel CAD | 51 (20.3%) | 147 (26.7%) |  |
| 2-vessel CAD | 73 (29.1%) | 151 (27.5%) |  |
| 3-vessel CAD | 127 (50.6%) | 236 (42.9%) |  |
| Culprit lesion |  |  | <0.001 |
| RCA | 66 (27.3%) | 99 (19.0%) |  |
| Left main | 2 (0.8%) | 18 (3.4%) |  |
| LAD | 67 (27.7%) | 202 (38.7%) |  |
| LCx | 76 (31.4%) | 93 (17.8%) |  |
| RIM | 3 (1.2%) | 5 (1.0%) |  |
| Bypass | 19 (7.9%) | 25 (4.8%) |  |
| not specified | 9 (3.7%) | 80 (15.3%) |  |
| Stenosis grade of culprit lesion |  |  | <0.001 |
| No Stenosis | 0 (0.0%) | 6 (1.1%) |  |
| <50% | 0 (0.0%) | 20 (3.6%) |  |
| 50-75% | 0 (0.0%) | 57 (10.4%) |  |
| 75-95% | 32 (12.7%) | 230 (41.8%) |  |
| 95-99% | 98 (39.0%) | 236 (42.9%) |  |
| 100% | 121 (48.2%) | 1 (0.2%) |  |
| TIMI flow grade of culprit vessel |  |  | <0.001 |
| 0 | 126 (50.2%) | 11 (2.0%) |  |
| 1 | 36 (14.3%) | 2 (0.4%) |  |
| 2 | 89 (35.5%) | 80 (14.5%) |  |
| 3 | 0 (0.0%) | 457 (83.1%) |  |
| *^1^n (%)* | | | |
| *^2^Fisher's exact test; Pearson's Chi-squared test* | | | |

NSTEMI: Non-ST-segment elevation myocardial infarction; OMI: Occlusion myocardial infarction; NOMI: Non-occlusion myocardial infarction; CAD: Coronary artery disease; RCA: Right coronary artery; LAD: Left anterior descending artery; LCx: Left circumflex artery; RIM: Ramus intermedius artery

**Supplemental Table 4.** Summary of the effects of each variable for time to coronary angiography

| **Variable** | **Low** | **High** | **Diff.** | **Effect** | **S.E.** | **Lower 0.95** | **Upper 0.95** |
| --- | --- | --- | --- | --- | --- | --- | --- |
| Age | 58 | 77 | 19 | 3.49 | 1.63 | 0.28 | 6.69 |
| Hs-cTnT at presentation | 29 | 163 | 134 | -10.67 | 2.05 | -14.7 | -6.64 |
| Sex – female:mlae | female | male | - | 6.58 | 2.41 | 1.85 | 11.32 |
| Previous Revascularisation | Yes | No | - | 3.0 | 2.06 | 1.04 | 7.03 |
| ST-depression | Yes | No | - | -5.90 | 2.22 | -10.26 | -1.52 |
| T-wave inversion | Yes | No | - | -1.28 | 2.22 | -5.65 | 3.08 |
| Morphin | Yes | No | - | -6.03 | 2.38 | -10.70 | -1.36 |
| Dyspnoe | Yes | No | - | 2.30 | 1.92 | -1.47 | 6.07 |
| Radiation to left shoulder | No | Yes | - | 1.13 | 1.90 | -2.59 | 4.85 |
| Chest pain characteristics – burning:pressure | burning | Pressure | - | -2.22 | 3.22 | -8.55 | 4.10 |
| Chest pain characteristics – sharp:pressure | Sharp | Pressure | - | -2.95 | 3.26 | -9.35 | 3.46 |
| Chest pain characteristics – ripping:pressure | ripping | Pressure | - | -8.97 | 4.75 | -18.31 | 0.36 |

Effect: difference in time to coronary angiography between both levels (e.g. patients with ST-segment depression underwent coronary angiography on average 5.9 hours earlier than patients without ST-segment depression.

**Supplemental Figure Legends**

**Supplemental Figure 1.** Partial (conditional) effects plots for the time to coronary angiography from ED presentation. The fitted linear regression models were conditioned for age, sex, previous revascularization, chest pain characteristics, ST-depression, T-wave-inversion, chest pain radiation to left shoulder/arm, dyspnoe, application of morphine and hs-cTnT concentration at presentation. For categorical variables the point represents the adjusted point estimate (i.e. time to coronary angiography), and the solid line represents the 95% confidence interval. For continuous variables (age and hs-cTnT) the solid line represents the adjusted point estimate (time to coronary angiography) and the shaded area indicates the 95% confidence interval.

**Supplemental Figure 2.** Association between time from presentation to coronary angiography (in hours) and the adjusted hazard ratio (adjHR) for (A) MACE and (B) cardiovascular death over 5 years for type I non-ST-segment elevation myocardial infarction (NSTEMI) patients with an occlusion myocardial infarction (OMI). The underlying cox proportional hazards model was adjusted for age, sex, GFR, diabetes mellitus, history of CAD and history of malignancy. The solid line represents the adjusted hazard ratio, and the shaded area indicates the 95% confidence interval. The dashed line at an adjHR of 1.0 serves as the reference, denoting no association. Time is modelled continuously, and the adjHR is calculated relative to the reference point of 12 hours’ time to coronary angiography. The box plot shows the patient distribution regarding time to coronary angiography (median 7.1 hours, interquartile range 4.7-22 hours).

**Supplemental Figure 3.** Association between time from presentation to revascularisation (in hours) and the adjusted hazard ratio (adjHR) for (A) all-cause death, (B) MACE and (C) cardiovascular death over 5-years for type I non-ST-segment elevation myocardial infarction NSTEMI patients with an occlusion myocardial infarction (OMI) who received revascularisation. Time to revascularisation is defined as time from presentation to coronary angiography for patients who received percutaneous coronary intervention (PCI) and time from presentation to surgery for patients who underwent bypass surgery. The underlying cox proportional hazards model was adjusted for age, sex, GFR, diabetes mellitus, history of CAD and history of malignancy. The solid line represents the adjusted hazard ratio, and the shaded area indicates the 95% confidence interval. The dashed line at an adjHR of 1.0 serves as the reference, denoting no association. Time is modelled continuously, and the adjHR is calculated relative to the reference point of 12 hours’ time to revascularisation. The box plot shows the patient distribution regarding time to revascularisation (median 7.7 hours, interquartile range 4.8-23.3 hours).

**Supplemental Figures**

**Supplemental Figure 1.** Partial (conditional) effects plots for time to coronary angiography from ED presentation.

**Supplemental Figure 2.** Association of time to coronary angiography and (A) 5-year MACE and (B) 5-year cardiovascular death

**A**

**B**

**Supplemental Figure 3.** Association of time to revascularisation and (A) 5-year all-cause death, (B) 5-year MACE and (C) 5-year cardiovascular death

**A**

**B**

**C**
